# Smartphone-based simplified Western blotting detection method

**DOI:** 10.17912/micropub.biology.002001

**Published:** 2026-03-24

**Authors:** Kai Aoki, Koyuki Ota, Kaoruko Watanabe, Honoka Ishii, Kazuhiro Takekoshi

**Affiliations:** 1 Department of Health and Nutrition, Faculty of Health Science, Niigata University of Health and Welfare, Niigata, 15, JP; 2 Institute of Medicin, University of Tsukuba, Tsukuba, 08, JP

## Abstract

Western blotting is a widely used technique for the detection of specific proteins and is a fundamental tool in biomedical research and education. However, conventional chemiluminescent detection systems are expensive and often inaccessible in educational or resource-limited laboratory environments. In this study, we evaluated a low-cost smartphone-based chemiluminescence detection method and demonstrated that it enables reliable qualitative detection of protein expression, highlighting its potential for both educational and basic research applications.

**Figure 1. Smartphone-based chemiluminescence imaging of Western blotting:**
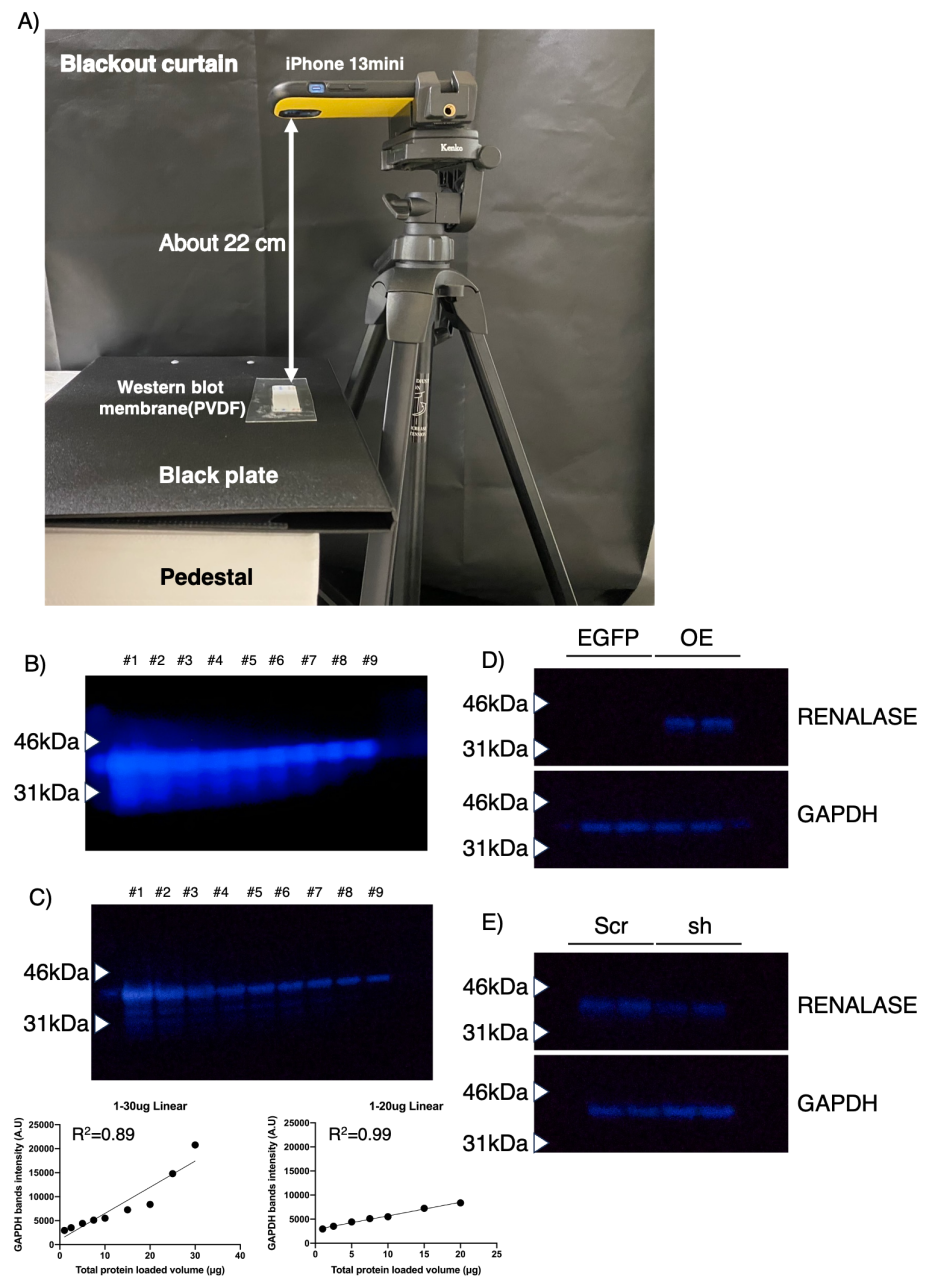
(A) Experimental setup for Western blot detection using a smartphone. (B) GAPDH detection using the native smartphone camera in night mode. Lanes #1–#9 correspond to total protein loads of 30, 25, 20, 15, 10, 7.5, 5, 2.5, and 1 μg, respectively. The lower graph shows the linear relationship between band intensity and total protein amount loaded. (C) Detection of GAPDH using an astrophotography application and confirmation of signal linearity. (D) Detection of renalase protein in renalase-overexpressing (OE) HCT116 cells. EGFP-expressing HCT116 cells were used as a control. (E) Detection of renalase following shRNA-mediated knockdown in renalase-OE cells. Scrambled shRNA (Scr)–expressing renalase-OE HCT116 cells served as a control, whereas renalase-specific shRNA (sh) indicates the knockdown condition.

## Description

Immunoblotting (Western blotting) is a globally established standard technique with a history spanning over 45 years [Moritz 2020]. The methodology is built upon foundational electrophoretic separation by sodium dodecyl sulfate–polyacrylamide gel electrophoresis (SDS-PAGE), originally described by Laemmli (1970), followed by electrophoretic transfer to membranes as reported by Towbin et al. (1979). The term “Western blotting” was later coined by Burnette (1981). Subsequent refinements of SDS-PAGE have further enhanced procedural efficiency and practical usability (Hagiwara 2022; 2025). Western blotting holds significant educational value, integrating multiple biochemical principles—including protein denaturation, electrophoretic separation, membrane transfer, and antigen–antibody interactions—and is widely incorporated into healthcare and science curricula (Begum 2022). However, the final step—chemiluminescence detection—often necessitates costly, dedicated imaging systems (e.g., CCD/CMOS cameras), priced from approximately JPY 1,980,000 (ATTO) to JPY 3,500,000 (BIO-RAD). This high cost renders implementation unaffordable for many institutions and classrooms, making the incorporation of Western blotting into educational courses challenging. In research settings, the lack of immediate equipment access creates a significant bottleneck for quickly ascertaining the qualitative presence of a protein or confirming the success of genetic modifications (knockout, knockdown, or overexpression). Recent advances in smartphone camera performance have facilitated the development of diverse biological applications, including fluorescence and luminescence detection, colorimetric and biosensing assays, quantitative protein analysis, and low-cost imaging or microscopy platforms (Hossain et al., 2017; Hosu et al., 2017; Kim et al., 2017; Krishnan et al., 2021; Moreira D.C., 2022; Moreira D.C., 2023; Cooper et al., 2023; Schaefer et al., 2023; Jiao et al., 2024; Yu et al., 2025). Therefore, we investigated whether chemiluminescence imaging could be successfully performed using ubiquitous smartphones, focusing on providing a highly accessible and practical detection alternative.

Initially, we tried to detect chemiluminescence signals using a standard smartphone camera with night mode. The smartphone was positioned as illustrated in Figure 1A to detect GAPDH. Although we could detect the GAPDH signals, band’s detail, shape and visibility was not satisfactory ( Figure 1B ). Based on this result, we next employed an application (app) designed for astronomical observation “AstroShader” due to its superior light capture capabilities compared to a standard camera application. GAPDH could be detected even with a total protein amount of 1 μg. Analyzing the linearity of the quantitative values obtained for GAPDH showed that the range from 1 μg to 30 μg demonstrated insufficient linearity for precise quantification (R ^2^ =0.89), while the restricted range from 1 μg to 20 μg exhibited high linearity (R ^2^ =0.99). We then verified the system's capability to detect overexpression and knockdown using the astronomical app when 10 μg of total protein was loaded ( Figure 1C, D). Renalase expression was confirmed in overexpressing HCT116 cells and was observed to be markedly fainter following shRNA-based knockdown (KD). This demonstrates that smartphones can be reliably used for the qualitative confirmation of protein overexpression and knockdown, providing a rapid and simplified checkpoint essential for many research protocols. However, since this experiment did not assess differences at physiological protein expression levels, nor did it detect knockdown or knockout at those endogenous levels, further investigation in this area is warranted.

To summarize our key findings: 1) GAPDH could be detected using the smartphone-based system, with a linear detection range from 1 μg to 20 μg of total protein loaded; 2) The system was capable of qualitatively detecting the presence or absence of a target protein; and 3) A clear reduction in protein level could be detected following shRNA-based KD treatments. Among low-cost alternatives, Khoury et al. successfully used a digital SLR camera (Khoury et al 2009). In contrast, our research aimed to explore an even simpler and more universally accessible methodology, leading us to select the use of a smartphone. Although smartphone camera performance varies across devices, the iPhone 13 mini successfully met the necessary detection thresholds. These findings suggest that detection efficacy depends more on the high-sensitivity, multi-exposure capabilities provided by specialized camera applications—such as those designed for astrophotography—rather than being limited to specific hardware models. This broad applicability is essential for educational purposes and rapid, on-site research. The detection parameters (exposure time, ISO, and number of exposures) were established as initial proof-of-concept settings based on standard chemiluminescence protocols, rather than exhaustive optimization. The system's primary advantage is its flexibility; users can easily adjust these settings to accommodate varying protein expression levels. In the future, smartphone-based platforms integrated with AI-driven analysis will offer cost-effective alternatives for diverse biological detections without requiring specialized laboratory equipment (Nonno and Ulber 2021; Avci 2025).In conclusion, although our method is not intended for highly precise quantitative analysis, we believe that it provides a practical and highly accessible solution for laboratories and educational environments lacking specialized, high-cost imaging equipment, allowing for the widespread implementation of Western blotting for fundamental understanding and rapid qualitative verification.

## Methods

Plasmids and Cell Culture

Caco-2 cells (Cat. No. 86010202) and HCT116 cells (Cat. No. 91091005) were purchased from ECACC (Salisbury, UK). Caco-2 cells were maintained in DMEM supplemented with 10% FBS, 1% MEM non-essential amino acids, and 1% penicillin-streptomycin. HCT116 cells were cultured in RPMI 1640 Medium supplemented with 10% FBS and 1% penicillin-streptomycin. All cells were incubated at 37 ∘C in 5% CO2​. Caco-2 cells were harvested after reaching 100% confluence. Plasmids (VectorBuilder) were transfected using FuGENE®4K. EGFP-HCT116 and renalase-overexpressing HCT116 overexpressing cell lines were established by puromycin selection (2 μg/mL) for 7 days, followed by single clone isolation. Cells expressing scrambled or renalase-specific shRNA were harvested three days after shRNA induction.

&nbsp;

Sample Preparation and Western Blotting

Total proteins were extracted using ice-cold 1% NP-40 lysis buffer (1% NP-40, 50 mmol/L Tris-HCl, 150 mmol/L NaCl, 1 mmol/L EDTA) supplemented with a protease inhibitor tablet (Roche, Basel, Switzerland). Cells were lysed, vortexed briefly, and centrifuged at 12,000 × g for 10 min at 4°C (Model 3530 centrifuge, KUBOTA CORPORATION). The supernatant was collected as whole-cell lysate. Protein concentrations were determined using a BCA protein assay kit (Nacalai Tesque). Samples were prepared in 2× sample buffer (Bio-Rad) containing 5% 2-mercaptoethanol and boiled prior to loading. For Figure 1B and C, 1–30 μg of total protein per lane was loaded. For Figure 1D and E, 10 μg of total protein per lane was separated on 12% SDS-polyacrylamide gels using SDS-PAGE running buffer (25 mmol/L Tris, 192 mmol/L glycine, 0.1% SDS). Polyacrylamide gels were prepared using a gel casting kit (Bio-Rad). Proteins were transferred onto polyvinylidene fluoride (PVDF) membranes using a Trans-Blot Turbo Transfer System with a corresponding transfer kit (Bio-Rad). Membranes were blocked with 5% skim milk in 0.1% TBS-T for 30 min at room temperature with agitation. Membranes were then incubated overnight at 4℃ with primary antibodies (anti-GAPDH or anti-Renalase) diluted in antibody dilution buffer (Beacle, Inc.), followed by washing three times for 5 min each with 0.1% TBS-T. Membranes were incubated with appropriate secondary antibodies diluted in 5% skim milk in 0.1% TBS-T for 1 h at room temperature with agitation. Immunoreactive bands were visualized using a chemiluminescent detection reagent (ATTO) after three additional washes. Detecting images were saved as RAW files. For quantification, RAW images were uploaded to PowerPoint, saved as PNG files, and then converted to TIFF files using ImageJ for subsequent quantification.

&nbsp;

Detection Conditions and Software

A dedicated detection setup was developed ( Figure 1A ) using an iPhone 13 mini mounted on a tripod. A pedestal (e.g., Styrofoam) was utilized to reduce the distance, and a black plate was placed beneath the membrane to suppress reflections. The distance between the membrane and the camera was set to approximately 22 cm. The detection area was enclosed by a blackout curtain to achieve the darkest environment possible. The astronomical observation application (AstroShader v1.0.34 (6)) was used for chemiluminescence detection. The key settings were configured as follows: focus 0.6, number of exposures 10, exposure time 5 sec, ISO 1597, camera timer 3 sec, and save format RAW.

## Reagents

Reagents

**Table:** 

CELL LINE	DESCRIPTION	MANUFACTURER	CAT. NO.
CACO-2	Human colorectal cancer cell line	ECACC	86010202
HCT116	Human colorectal cancer cell line	ECACC	91091005
PLASMID	CONSTRUCT	MANUFACTURER	CAT. NO.
pRP[Exp]-EGFP/ Puro-CAG>ORF_stuffer	EGFP/Puromycin	Vector Builder	VB010000-9288rhy
pRP[Exp]-EGFP/Puro-CAG>hRNLS[NM_001031709.3]	EGFP/Puromycin Human Renalase	Vector Builder	VB900015-4260jwp
pRP[shRNA]-EGFP/ Puro-U6>Scramble[shRNA#1]	EGFP/Puromycin Scramble shRNA	Vector Builder	VB010000-9341vde
pRP[shRNA]-EGFP:P2A:Puro-U6>hRNLS[shRNA#1]	EGFP/Puromycin Human Renalase specific shRNA	Vector Builder	VB250723-1661cfb
REAGENTS	DESCRIPTION	MANUFACTURER	CAT. NO.
FuGENE®4K	Transfection reagents	Promega	E5911
Puromycin	Selection antibiotics	INVIVOGEN	ant-pr-1
MEM Non-Essential Amino Acids Solution(100x)	NEAA solution	Nacalai Tesque	06344-56
Penicillin-Streptomycin Solution (×100)	Antibiotics	WAKO	168-23191
cOmplete™, Mini	Proteinase inhibitor	Roche	04693124001
Protein Assay BCA Kit	Quantification of protein concentration	Nacalai Tesque	06385-00
2×Laemmli Sample Buffer	Preparation of loading sample	Bio-Rad	#1610737
TGX™ FastCast™ Acrylamide Solution Kit, 12%	Acrylamide gel casting kit	Bio-Rad	#1610175
Transblot Turbo RTA Transfer Kit PVDF (Mini)	Transfer kit including PVDF membrane and buffer	Bio-Rad	# 1704272
Tris Buffered Saline with Tween ^®^ 20 (TBS-T) Tablets, pH7.6	Wash buffer	Takara	T9142
EzWestLumi Plus	HRP luminescent substrate	ATTO	WSE-7120S
Signal Booster	Antibody diluent reagent	Beacle Inc.	BCL-125A
ANTIBODY	ANIMAL AND CLONALITY	DESCRIPTION	
Anti-GAPDH	Mouse monoclonal	Santacruz Biotechnology, sc-365062, Dilution ratio 1:500	
Anti-Renalase	Rabbit polyclonal	Proteintech Group, 15003-1-AP, Dilution ratio 1:2000	
HRP conjugate secondary antibody	anti-rabbit or mouse IgG	CST, 7074 or 7076, Dilution ratio 1:5000	
